# Does repetition increase perceived truth equally for conspiracy and trivia statements? A registered replication report

**DOI:** 10.3758/s13423-025-02836-w

**Published:** 2026-01-07

**Authors:** Shauna M. Bowes, Lisa K. Fazio

**Affiliations:** 1 Vanderbilt University, Nashville, TN USA; 2 Psychology Department, The University of Alabama in Huntsville, 301 Sparkman Drive, Room 206, Huntsville, AL 35899 USA

**Keywords:** Illusory truth, Conspiracy belief, Conspiracy theory, Repetition effects

## Abstract

Repetition increases the perceived truth of information. This illusory truth effect is a well-documented and robust phenomenon. Although research has primarily focused on trivia statements, the effects of repetition on belief have also been identified for consequential statements such as fake news headlines. Moreover, research reveals repetition increases accuracy ratings for conspiracy statements. However, in past work, the illusory truth effect was smaller for conspiracy statements than trivia statements. This result raises the intriguing possibility that there is something unique about conspiracy statements relative to trivia statements that makes them more resistant to the effects of repetition. However, this difference in the illusory truth effect between conspiracy and trivia statements may be due to differences in baseline plausibility rather than anything specific about conspiracy statements. Overall, the conspiracy statements were seen as less plausible than the trivia statements (both true and false trivia statements) in the prior experiment. In this registered report, we examined the illusory truth effect for conspiracy and trivia statements using the same procedure as in previous research, but we matched the statements on baseline plausibility. In line with our hypothesis, the effect of repetition on perceived truth was similar for conspiracy and trivia statements when they were equally implausible (or plausible). Results from this study replicate the generality of the illusory truth effect to statements that can cause harm and suggest that the psychological effect of repetition on truth ratings is equivalent for equally implausible (or plausible) conspiracy and trivia statements.

**Supplementary Information:**

The online version contains supplementary material available at 10.3758/s13423-025-02836-w.

A robust literature indicates that repetition increases perceptions of truth, a phenomenon largely referred to as the illusory truth effect (Dechêne et al., 2010; see Unkelbach et al., 2019). This effect of repetition on perceived truth is consistent and reliable, manifesting across ages (Brashier et al., 2017; Fazio & Sherry, 2020), even when statements contradict prior knowledge (Fazio et al., 2015) or are highly implausible (Fazio et al., 2019; Lacassagne et al., 2022), and following a delay as long as 1 month (Henderson et al., 2021). The illusory truth effect has most often been studied for general knowledge and trivia statements (Dechêne et al., 2010; Henderson et al., 2022), but the illusory truth effect has also been found where increases in belief can cause real harm such as for false health claims (Vellani et al., 2023) and fake news headlines (Pennycook et al., 2018; Pillai et al., 2023).

These results raise the possibility that repetition also increases belief for other types of harmful statements such as conspiracy theories. Conspiracy theories are causal statements advancing that a small, powerful group is acting in secret to harm the common good and benefit themselves (e.g., Uscinski, 2019). Belief in conspiracy theories is pervasive, with most Americans endorsing at least one conspiracy theory (e.g., Oliver & Wood, 2014). Even seemingly fringe conspiracy theories (e.g., lizard people secretly control global politics) may be seen as at least possibly true by millions of Americans (Bump, 2013). Repeated exposure to one conspiracy theory may contribute to that conspiracy theory being processed more fluently, thus increasing belief in that conspiracy theory (e.g., Clifford & Sullivan, 2023; see Udry & Barber, 2023). Moreover, repeated exposure to one conspiracy theory may lead to belief in other conspiratorial frames, as belief in one conspiracy theory often increases acceptance of other conspiracy theories (e.g., Swami et al., 2011). Believing in one conspiracy theory lowers the threshold of plausibility for other conspiracy theories, even those that seem unrelated, and makes people more likely to believe multiple conspiracy theories (e.g., Swami et al., 2011; Williams et al., 2022; van Prooijen & Douglas, 2018). Altogether, repeated exposure to a conspiracy theory may be one mechanism by which conspiracy belief increases.

To our knowledge, just one study has examined whether there is an illusory truth effect for conspiracy theories (Béna et al., 2023). In this study, the illusory truth effect was examined for false conspiracy statements and true and false trivia statements. In line with the larger literature on the illusory truth effect (e.g., Udry & Barber, 2023), repeated statements were judged as more accurate than new statements for both trivia and conspiracy statements. That said, there was a significant interaction between repetition and statement type, as the effect of repetition was approximately four times larger for trivia statements than for conspiracy statements. The magnitude of the illusory truth effect was not moderated by conspiracy mentality (i.e., general tendencies to believe in conspiratorial statements; Bruder et al., 2013) or cognitive reflection (i.e., tendencies to override intuition and engage in analytical thinking; Frederick, 2005). Overall, this study provides evidence for an illusory truth effect for conspiracy theories, and it indicates that

1. The effect of repetition on perceived truth is smaller for conspiracy theories than for trivia statements and
2. Is not moderated by relevant individual differences variables.

If replicated, this difference between conspiracy theories and trivia statements is interesting because it would clarify boundaries on the illusory truth effect. That is, such a difference would mean that the illusory truth effect does not generalize to the same extent to conspiratorial statements and that there is something potentially unique about interpretations of conspiratorial statements relative to trivia statements. Indeed, emerging research reveals that the magnitude of the illusory truth effect may be smaller, albeit still robust, for news headlines and health claims compared with trivia statements (Pennycook et al., 2018; Pillai et al., 2023; Vellani et al., 2023). Such results raise the possibility that the effects of repetition on perceived truth are strongest for trivia statements relative to other types of statements. In addition to processing fluency, other factors, such as prior knowledge or partisanship, may constrain the magnitude of the illusory truth effect for news headlines, health claims, or conspiratorial statements compared with trivia statements.

However, the differences in effect size across types of statements may be due to other factors. Specifically, previous research has not directly compared the illusory truth effect for trivia statements to the illusory truth effect for conspiracy statements when matching for baseline plausibility. Although the illusory truth effect occurs across different levels of plausibility, the observed repetition effect is smallest for highly implausible and highly plausible statements relative to moderately plausible statements (Fazio et al., 2019; Pennycook et al., 2018). In other words, when statements are perceived to be extremely inaccurate (or accurate), there is restricted variance in baseline perceptions of truth that in turn restricts the magnitude of the illusory truth effect. Thus, differences in the illusory truth effect between trivia statements and other types of statements may be due to that fact that the truth status of the selected trivia statements is ambiguous (Dechêne et al., 2010) whereas the truth status of other types of statements is clear (either seen as plausible or implausible).

In the previous study on repetition and conspiracy statements (Béna et al., 2023), the illusory truth effect may have been smaller for conspiracy statements because they were viewed as less plausible than the trivia statements, and ratings were farther from the midpoint. Put differently, the psychological effect of repetition may be the same for conspiracy and trivia statements, but they appear to be different effects simply due to differences in baseline plausibility. In fact, in the prior study there was a significant main effect of statement type such that both true and false trivia statements were, in general, seen as more accurate than conspiracy statements^1^ (Béna et al., 2023). Additionally, the truth ratings for the trivia statements were closer to the midpoint of the rating scale than the truth ratings for conspiracy statements (Béna et al., 2023).

As such, in this registered report, we aim to replicate and extend upon these findings by examining the illusory truth effect for conspiracy statements and trivia statements when they are matched for initial accuracy. In so doing, we will clarify the extent to which repetition contributes to increased conspiracy belief and whether there are boundaries to the illusory truth effect when examining different types of statements. We hypothesize that the magnitude of the illusory truth effect will not significantly differ across equally implausible (or plausible) conspiracy statements and trivia statements. We directly replicate the methodology from Béna et al. (2023) but use trivia and conspiracy statements that are matched for baseline plausibility.

## Method

All data, materials, supplemental analyses, and code are publicly available on the Open Science Foundation (OSF) repository (https://osf.io/b8hjk/overview).

### Participants

Participants were recruited via CloudResearch Connect. All participants were American adults, and participants who completed the pretest (described in “Materials”) were not eligible to participate in the study. We aimed to match the sample size reported in Béna et al. (2023; *N* = 282). A sensitivity analysis in G*Power (Faul et al., 2009) revealed that this sample size provides 89% power to detect the small effect of repetition on truth ratings for conspiracy statements reported by Béna et al. (2023; *d*_z_ = .17). As preregistered, data collection was terminated once 296 participants completed the study (which accounts for 5% of participants potentially having invalid data); 297 participants ultimately completed the survey due to over-recruitment by CloudResearch. Upon screening the data, we removed data from one participant who did not pass both attention checks (described in “Procedure”) for a final sample size of 296 participants (*M*_age_ = 38.46 years, *SD*_age_ = 12.09). Most participants were male (51.0%), White (72.0%), not Latino (87.5%), Christian (54.1%), and politically Democratic (47.6%).

### Materials

#### Conspiracy and trivia statements

We created a set of 80 conspiracy statements that we subjected to a pretest on CloudResearch Connect (*N* = 96). The pretest data, materials, and results are on the OSF repository. All conspiracy statements were pulled from existing measures/surveys (Bump, 2013; Enders & Uscinski, 2021; Enders et al., 2021; Federico et al., 2018; Miller et al., 2016; Shapiro et al., 2016; Swami et al., 2011; Uscinski et al., 2022; van Prooijen et al., 2023) or created because they were not yet included on existing measures but were relevant to the current sociopolitical context (e.g., Maui fires, Federal Emergency Management Agency nationwide alert). Participants were randomly shown 40 of the 80 conspiracy statements, and they indicated how accurate each statement was on a 1 (*definitely false*) to 6 (*definitely true*) scale.^2^ We computed the mean accuracy rating and standard deviation for each conspiracy statement.

We compared the average accuracy ratings (the means) for the conspiracy statements to the average accuracy ratings for 80 false trivia statements used in previous research that were rated on the same 6-point false–true scale by 128 participants (Fazio, 2020). We matched conspiracy statements to trivia statements in two ways. First, conspiracy statements were matched to trivia statements where there was a .05 or smaller difference between the average plausibility ratings. Second, we sought to maximize item diversity for the conspiracy statements and not overrepresent a particular domain (e.g., vaccine conspiracy theories). Thus, after matching conspiracy statements to trivia statements based on the mean difference score, we pruned the set of statements to include a diverse range of existing conspiracy theories (e.g., vaccine conspiracy theories, political conspiracy theories).

After completing these two steps, we created two sets of matched statements (Set A and Set B), with each set comprising of 18 conspiracy statements and 18 trivia statements (see Table 1 for example matched pairs). Thus, there were 36 conspiracy statements and 36 trivia statements total. The average mean truth ratings and standard deviations were similar across the two sets (Set A: *M* = 2.61, *SD* = .63, range: 1.60–4.03; Set B: *M* = 2.62, *SD* = .53, range: 1.62–3.56). The average mean difference across matched conspiracy and trivia statements was .02 for both statement sets (and the standard deviation of these difference scores was .01 for both statement sets). The final set of matched statements, means, and differences across means is on the OSF repository.^3^

**Table 1:** Example matched conspiracy statement and trivia statement pairs

	Statement	Mean accuracy rating
**Conspiracy**	Tiny devices are implanted in vaccines for use in mind control experiments	1.62
**Trivia**	A hurricane is a cyclone that occurs over land	1.65
**Conspiracy**	The 2007–2008 financial crisis was secretly orchestrated by a small group of Wall Street bankers to extend the power of the Federal Reserve and further their control of the world's economy	2.64
**Trivia**	Anthony is the last name of the founder of the American Red Cross	2.65
**Conspiracy**	The assassination of John F. Kennedy was not committed by the lone gunman, Lee Harvey Oswald, but was rather a detailed, organized conspiracy to kill the president	3.02
**Trivia**	Gotti is the criminal who was known for “Scarface”	3.00

#### Individual differences measures

Consistent with the previous study on the illusory truth effect for conspiracy statements (Béna et al., 2023), participants completed two individual differences measures: general conspiracy belief and cognitive reflection. In order to increase our study’s power to detect individual differences, we used longer versions of each scale as compared with Béna et al. (2023). To measure general conspiracy belief, participants completed the 15-item Generic Conspiracist Beliefs Scale (GCBS; Brotherton et al., 2013). Participants indicated their agreement with each statement on a 1 (*completely false*) to 9 (*completely true*) scale. Items were summed to create a total conspiracy belief score (*M* = 68.05, *SD* = 31.09; α = .96). Note, items from the GCBS were not part of the conspiracy statement pretest and do not overlap with our conspiracy statements.

Cognitive reflection was measured with eight fill-in-the-blank questions where there is an intuitive answer that must be overridden to provide the correct answer (Meyer et al., 2024). Correct answers were scored as “1” and incorrect answers were scored as “0”. Correct answers were summed to create a total cognitive reflection score (*M* = 4.28, *SD* = 2.73; α = .85).

#### Demographics

Participants indicated their gender, level of education, race, ethnicity, age, political identification, political belief strength, and religious affiliation. Note, the demographic information was collected to characterize the sample rather than to be used as moderator or control variables. The specific response options for each of the demographic variables are included on the OSF platform.

### Design

Matching Béna et al. (2023), we used a 2 (repetition: new, repeated) × 2 (statement type: conspiracy, trivia) within-subjects design. We manipulated statement type within-subjects to replicate the Béna et al. study. In addition, a within-subjects design is ostensibly more ecologically valid than a between-subjects design, given that individuals encounter a range of statement types in their information environments as opposed to just one statement type. Thus, we sought to clarify the effects of repetition on perceived truth when individuals simultaneously evaluate different types of statements, in this case conspiracy and trivia statements.

### Procedure

Before participating in the study, participants were asked two attention check questions. One of these questions asked participants to make two selections from a multiple-choice list, and the second question asked participants to complete a fill-in-the-blank task. If participants failed both attention checks, they were bounced from the survey. If participants passed at least one attention check, they moved forward in the survey.

Participants then began the exposure phase. For counterbalancing purposes, participants were randomly assigned to view one of the two matched statement sets (A or B, each of which comprises 18 conspiracy statements and 18 trivia statements) for the exposure phase. Participants indicated their level of interest in each of 36 statements (presented individually) on a 1 (*very uninteresting*) to 6 (*very interesting*) scale. Statements were presented in a random order with the conspiracy and trivia statements intermixed (as in Béna et al., 2023).

Immediately after this exposure phase, participants completed the truth rating phase. We adopted the instructions from the Béna et al. (2023) study for the truth rating phase (which are provided on the OSF page); the only deviation from their instructions is that we did not tell participants that some statements are true and some are false, given that we only included false trivia statements. Participants saw the full set of 72 statements with both conspiracy statements and trivia statements (each statement presented individually) and rated the accuracy of each statement on a 1 (*definitely false*) to 6 (*definitely true*) scale.^4^ The order of the statements was randomized across participants.

After the truth rating phase, participants completed the individual differences measures (conspiracy belief first and then cognitive reflection) and finally the demographic measures. They then saw the debrief and finished the study.

## Results

### Illusory truth effect

We first examined the magnitude of the illusory truth effect across conspiracy and trivia statements by conducting a preregistered 2 (repetition: repeated, new) × 2 (materials: conspiracy statements, trivia statements) within-subjects analysis of variance (ANOVA) on the average truth rating scores. Consistent with previous research on the illusory truth effect (e.g., Béna et al., 2023; Fazio et al., 2019), repeated statements were given higher truth ratings than new statements, *F*(1, 295) = 29.32, *p* < .001, partial η^2^ = .09. Repetition significantly increased truth ratings for both conspiracy statements (*M*_new_ = 2.83, *SD*_new_ = .98; *M*_repeated_ = 2.91, *SD*_repeated_ = 1.00); *t*(295) = 3.14, *p* = .002; *d*_z_ = .18, and trivia statements (*M*_new_ = 2.80, *SD*_new_ = .85; *M*_repeated_ = 2.95, *SD*_repeated_ = .99); *t*(295) = 4.47, *p* < .001; *d*_z_ = .26. In line with our hypotheses, the main effect of materials on truth ratings was not statistically significant, *F*(1, 295) = .01, *p* = .928, partial η^2^ < .001, and this was true for both new, *t*(295) = .60, *p* = .548; *d*_z_ = .04, and repeated, *t*(295) = .72, *p* = .474; *d*_z_ = .04, statements.

**Fig. 1:**
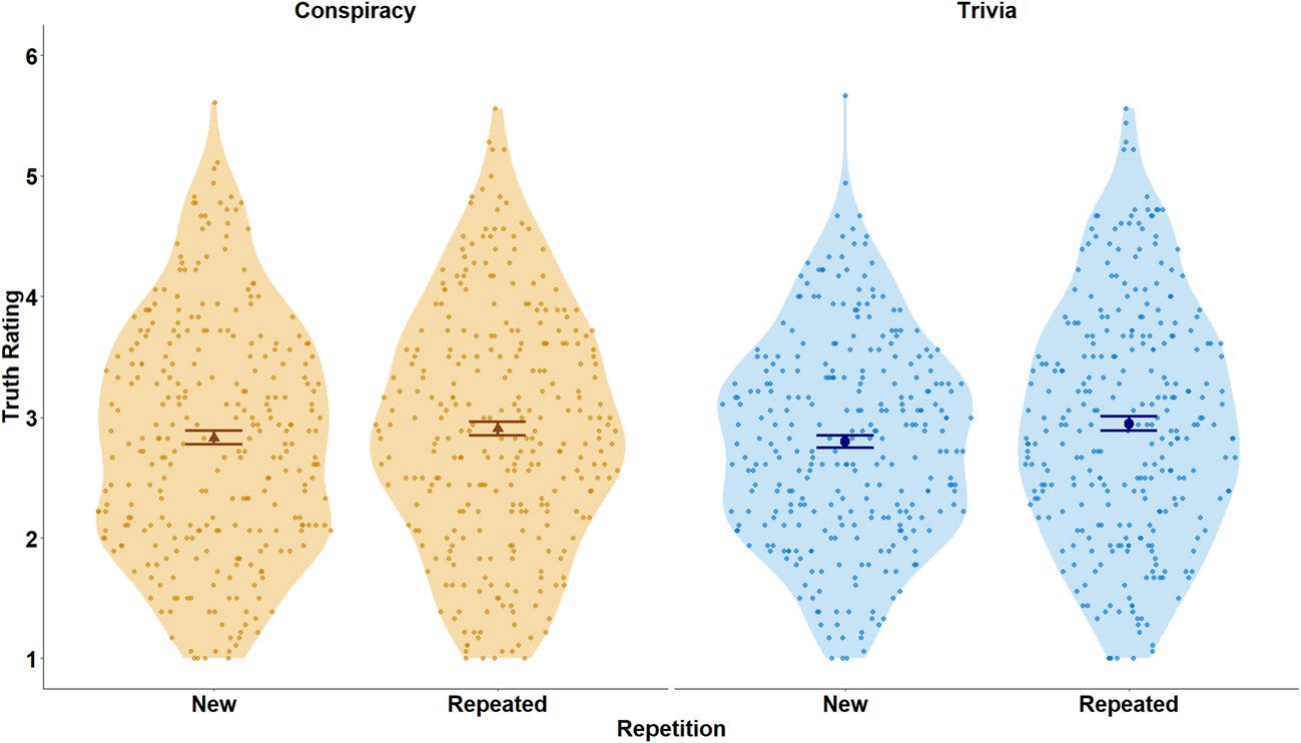
Mean truth ratings for conspiracy and trivia statements by repetition status. *Note.* Ratings were provided on a scale from 1 (*definitely false*) to 6 (*definitely true*). Each dot represents one participant (*N* = 296). The triangles reflect group means for the conspiracy statements, and the circles reflect group means for the trivia statements. The error bars reflect standard errors of the mean

Our key hypothesis was that there would be no interaction between repetition and materials, and this was confirmed, *F*(1, 295) = 3.53, *p* = .061, partial η^2^ = .01.^5^ The magnitude of the illusory truth effect did not significantly differ between conspiracy and trivia statements (Fig. 1). In secondary analyses that were not pre-registered, we used mixed-effects regressions to examine the effects of repetition, statement, and their interaction on truth ratings for item-level truth ratings with participant and item random intercepts. These results are described in detail in the “Supplemental Materials” document on the OSF repository (Supplemental Materials 1; Supplemental Fig. 1). To summarize, we found a very small, but significant, interaction between repetition and materials, *t*(20,940) = 2.01, *p* = .044, *b* = .08, *SE* = .04, 95% CI [.00, .15], such that the magnitude of the illusory truth effect was slightly higher for trivia statements than for conspiracy statements.

Given that the effect in both analyses was small and close to the .05 alpha level, we conducted a (non-preregistered) follow-up Bayesian analysis to quantify the amount of support for the null hypothesis that the effects of repetition were similar for both conspiracy and trivia statements. Using JASP (Version 0.18.2; JASP Team, 2023) and a Bayesian repeated-measures ANOVA, we computed a Bayes factor for the repetition-by-materials interaction (i.e., the relative predictive performance of the null hypothesis versus the alternative hypothesis; Keysers et al., 2020). We focus on the BF_inclusion_ for the interaction term, which was computed using the matched model method. The BF_inclusion_ for the interaction term is the average probability of a model given the data for all models that include the interaction term divided by the average probability of a model given the data for the matched models that are only missing the interaction term. A Bayes factor of 1 indicates that the observed data are equally likely to occur under the models with and without the interaction term, a Bayes factor >1 indicates that the observed data are more likely to occur under the models with the interaction term than without the interaction term, and a Bayes factor of <1 indicates that the observed data are more likely to occur under the models without the interaction term than with the interaction term (see Keysers et al., 2020). We found weak evidence against including the interaction term (BF_inclusion_ = .58). The data were 1.72 times more likely under the models without the interaction term than with the interaction term. Thus, these analyses provide additional evidence that there is not a meaningful repetition-by-materials interaction.

### Individual differences moderators

Consistent with the previous study on the illusory truth effect for conspiracy statements (Béna et al., 2023), we examined whether conspiracy belief and cognitive reflection moderate the illusory truth effect using mixed-effects regression analyses and the *lme4* (Bates et al., 2015) package in R Studio. We used the methods reported in Béna et al. (2023)—the truth rating variable for each item was the dependent variable, and both participant and item were included as random intercepts. We examined the main effects of repetition (dummy-coded, repeated or new), materials (dummy-coded, conspiracy or trivia), and conspiracy mentality or cognitive reflection in addition to all potential two-way interactions and the three-way interactions between repetition, materials, and conspiracy mentality or cognitive reflection in this preregistered analysis.^6^ Full results are reported on the OSF platform.

#### Conspiracy belief

**Fig. 2:**
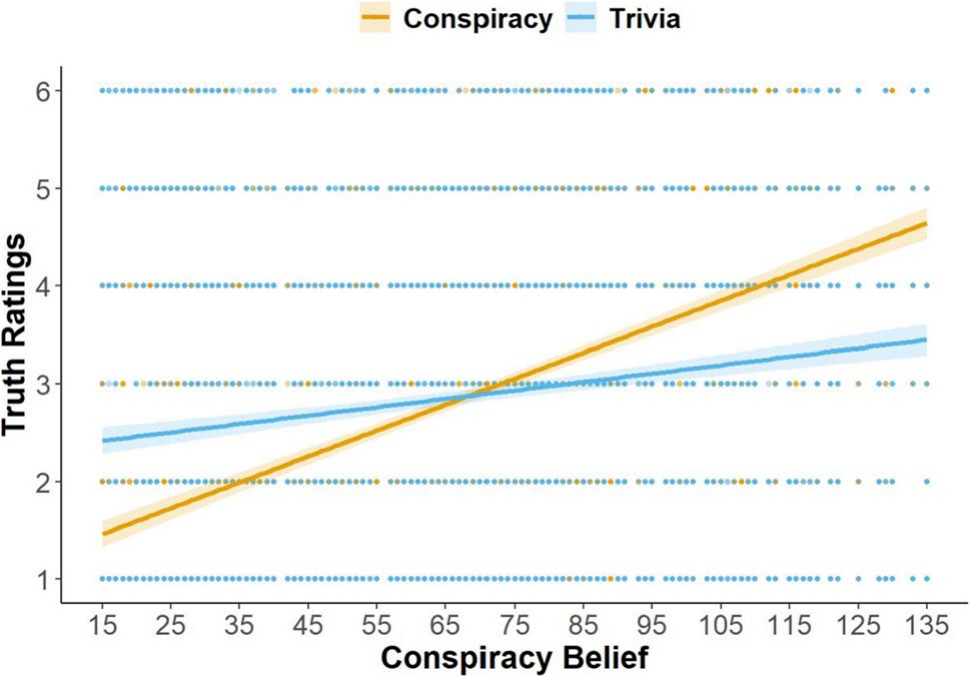
Interaction between conspiracy belief and materials in predicting truth ratings. *Note.* Scores on the *x*-axis reflect sum scores on the Generic Conspiracy Belief Scale. Error bars reflect 95% confidence intervals around the predicted regression lines. (Color figure online)

Results are detailed in Supplemental Table 1. The main effects of materials (*b* = 1.22, *p* < .001) and conspiracy belief (*b* = .03, *p* < .001) on truth ratings were significant such that (1) trivia statements were rated as more true than conspiracy statements and (2) conspiracy belief was related to higher truth ratings. Crucially, however, these main effects were qualified by a significant two-way interaction between materials and conspiracy belief (*b* = −.02, *p* < .001). Simple slopes analyses revealed that conspiracy belief was significantly related to higher truth ratings for conspiracy (*b* = .03, *p* < .001) and trivia statements (*b* = .01, *p* < .001), but the relationship was stronger for conspiracy than for trivia statements (Fig. 2). None of the other main effects or interaction terms were statistically significant.

#### Cognitive reflection

**Fig. 3:**
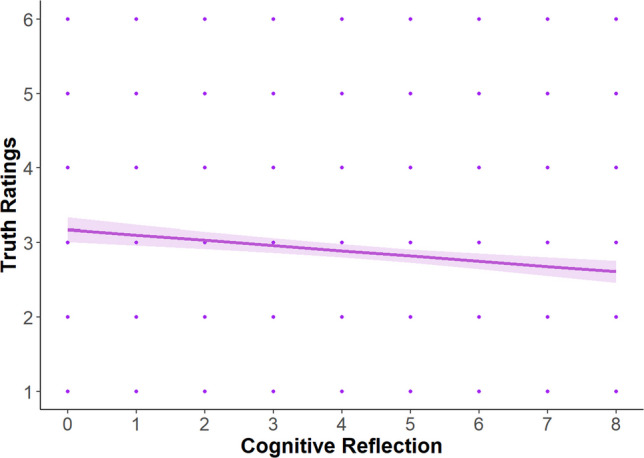
Main effect of cognitive reflection on truth ratings. *Note.* Scores on the *x*-axis reflect sum scores on the Cognitive Reflection Test. The error bar reflects the 95% confidence interval around the predicted regression line

Results are detailed in Supplemental Table 2. The main effect of cognitive reflection (*b* = −.06, *p* < .001) on truth ratings was significant such that more cognitive reflection predicted lower truth ratings (Fig. 3). None of the other main effects or interaction terms were statistically significant.^7^

## Discussion

Previous research found that the magnitude of the illusory truth effect was smaller for conspiracy statements than for trivia statements (Béna et al., 2023). However, in that study, trivia and conspiracy statements were not matched for baseline plausibility. Given that baseline plausibility affects the magnitude of the illusory truth effect (e.g., Fazio et al., 2019), it is not possible to ascertain whether the effect of repetition on perceived truth differs between conspiracy and trivia statements (a point also noted by the original authors). As such, we sought to replicate and extend prior research by examining whether the magnitude of the illusory truth effect varied across conspiracy and trivia statements when they were matched for baseline plausibility.

Replicating the larger literature on the illusory truth effect (see Udry & Barber, 2023), repeated statements were rated as more true than new statements, for both conspiracy and trivia statements. Our main hypothesis centered around the interaction between repetition and materials. We hypothesized that the magnitude of the illusory truth effect would be similar for equally implausible (or plausible) conspiracy and trivia statements. Consistent with our hypothesis, there were no significant differences in the magnitude of the illusory truth effect between conspiracy and trivia statements when matching statements based on baseline plausibility in our preregistered analyses. Note that in exploratory mixed-effects regressions we did find a small interaction between repetition and materials. Nevertheless, even in the mixed-effects regressions, which accounted for item-level variability, the effect size was still small. Based on these results in total, when eliminating confounds due to differences in baseline plausibility, the effect of repetition on perceived truth is similar for conspiracy statements and false trivia statements.

These results do not imply that conspiracy statements are semantically or psychologically equivalent to trivia statements. After all, prior knowledge, social identity, directional motivations, emotionality, sociopolitical context, and more would differentially predict belief in conspiracy statements and trivia statements. Conspiracy and trivia statements also make dramatically different types of claims (e.g., that small powerful groups operate in secret to harm the common good vs. general knowledge about the world). Although these differences are notable, they do not change how repetition affects belief. Instead, our results illuminate that the effect of repetition on belief is consistent across types of claims when their truth status is equally ambiguous (or clear). Our findings provide further evidence that the illusory truth effect is a low-level cognitive process that is unaffected by social cues and higher-level cognition (e.g., Pillai & Fazio, 2024).

We also examined whether conspiracy belief and cognitive reflection significantly moderated the effects of repetition on truth ratings across statement types. In line with previous research (Béna et al., 2023), neither conspiracy mentality nor cognitive reflection influenced the magnitude of the illusory truth effect across conspiracy and trivia statements. This finding is also broadly consistent with the larger literature on the illusory truth effect, as previous studies reveal that there are not robust individual differences predictors or moderators of the magnitude of the illusory truth effect (e.g., De Keersmaecker et al., 2020). We did find one significant moderator: those who score high on conspiracy belief are more likely to see both trivia and conspiracy statements as true, but the effect is especially strong for conspiracy statements relative to trivia statements.

### Limitations

Despite our study’s strengths (e.g., including a diverse range of conspiracy statements, matching conspiracy statements and trivia statements based on baseline plausibility ratings), there are limitations that should be considered when interpreting our findings. First, we were not sufficiently powered to detect an interaction between repetition and materials as small as the observed effect of η^2^ = .01; post hoc sensitivity analyses using *Superpower* in R revealed that we only had 41% power to detect the identified moderation effect. However, even if the interaction effect was statistically significant in a well-powered study, it is unlikely to be practically significant. The semantic, psychological, and plausibility differences between conspiracy statements and trivia statements do not strongly or substantively alter the effects of repetition on belief.

In addition, we used a within-subjects design

1. To directly replicate the methodology used in Béna et al. (2023) and
2. Because a within-subjects design is ostensibly more ecologically valid than a between-subjects design, given that people simultaneously encounter a range of different types of statements in their information environments.

Using a between-subjects design allows one to answer different questions than using a within-subjects design. In a between-subjects design, the following question can be answered: When exposed to conspiracy (trivia) statements, what is the effect of repetition on perceived truth? In a within-subjects design, the following question can be answered: When being exposed to a mixture of statements, what is the effect of repetition on perceived truth? We were primarily interested in the latter question, as we aimed to clarify how repeated conspiracy and trivia statements were processed relative to the average processing fluency for both types of statements. Future research can use a between-subjects design to answer different questions about the nature of the illusory truth effect and to examine the robustness of the findings across methods.

### Future directions

People are often repeatedly exposed to conspiracy theories in their information ecosystems, either due to bad actors or even accurate reporting of current sociopolitical events. This repeated exposure to conspiracy theories could be one situational mechanism by which conspiracy belief increases over time. Since our study did not include a longitudinal component, future research should examine whether the effects of repetition on perceived truth for conspiracy statements persist over time.

Similarly, future research should examine whether increased belief in one conspiracy theory translates to increased belief in other conspiracy theories. As mentioned earlier, believing one conspiracy theory predicts believing other, even unrelated, conspiracy theories, as the threshold of plausibility for other conspiracy theories is lowered after initial acceptance of a conspiracy theory (e.g., Williams et al., 2022). Thus, repeated exposure to one conspiracy theory may increase belief in both that theory and acceptance of other conspiratorial frames. Adopted conspiracy theories can coalesce into a monological belief network that is self-reinforcing and difficult to change (e.g., Williams et al., 2022). Research is needed to clarify whether repetition is a situational mechanism by which *overall* levels of conspiracy belief increase (as opposed to just for the specific conspiratorial frames that were repeated).

It will also be important to examine whether the illusory truth effect for conspiracy statements influences not only belief in conspiracy statements but also intentions and behaviors. For instance, repetition also affects moral reasoning—people view sharing fake news as less unethical when the headlines are repeated (e.g., Effron & Raj, 2020), and moral wrongdoings are seen as less immoral when they are repeated (e.g., Pillai et al., 2023). Repetition also increases belief in and intentions to share false health claims (Vellani et al., 2023). Increased belief in conspiracy theories could lead to an increase in sharing of conspiracy theories, which may contribute to their spread and amplify the repetition effect on belief in conspiracy statements. Research should directly examine whether repetition affects willingness to share conspiracy theories and taking action based on conspiracy theories. Such research would shine a light on the potential dangers of repeating conspiracy theories beyond increasing belief in conspiracy theories.

## Supplementary Information

Below is the link to the electronic supplementary material.

Supplementary file1 (DOCX 24 KB)

## Data Availability

All data and materials are available on the OSF repository: https://osf.io/b8hjk/?view_only=85807ad9bdf649078316a4284a3424ec

## Footnotes

1 Béna et al. (2023) did not report the means for accuracy ratings separately by true and false trivia statements. We, thus, examined these means using their publicly available dataset. Specifically, we conducted paired-samples *t* tests to compare the mean accuracy ratings for true and false trivia statements to each other and to the mean accuracy ratings for conspiracy statements, both when the statements were new and when they were repeated. All *t* statistics were significant per two-tailed *p* values (*t*s ranged from 3.96 [true versus false trivia statements, repeated] to 29.02 [true trivia statements versus conspiracy statements, repeated], *p*s < .001). They revealed that false trivia statements were rated as less accurate than true trivia statements (new: *d*_*z*_ = .28; repeated: *d*_*z*_ = .23), and conspiracy statements were rated as less accurate than false trivia statements (new: *d*_*z*_ = 1.19; repeated: *d*_*z*_ = 1.46) and true trivia statements (new: *d*_*z*_ = 1.52; repeated: *d*_*z*_ = 1.68). So, irrespective of statement truth for the trivia statements, conspiracy statements were seen as less accurate than trivia statements. The differences in truth ratings between conspiracy statements and trivia statements were not driven solely by higher truth ratings for true trivia statements.

2 Participants also indicated their level of familiarity with each conspiracy statement on a 1 (*definitely unfamiliar*) to 6 (*definitely familiar*) scale and how negative each statement was on a 1 (*extremely negative*) to 6 (*extremely positive*) scale. We also collected demographic information for gender, education, ethnicity, race, socioeconomic status, age, political party, and religious affiliation. We do not focus on these measures here, but the means and standard deviations for these ratings for each conspiracy statement as well as the demographic information are available on the OSF repository (alongside the Qualtrics file).

3 We also examined a range of additional descriptive statistics across the two types of statements. The distributions for each item across the two types of statements were similar according to different graphs and statistics (histograms, Q–Q plots, Kolmogorov–Smirnov test statistics, Shapiro–Wilk’s test statistics, average skewness statistics). There was also no systematic evidence for bimodal distributions across types of statements. In addition, the medians and modes for each item were similar across the two types of statements (medians tended to be ratings of 1 or 2; modes tended to be ratings of 1). The full range of possible ratings was captured for nearly all conspiracy statements and trivia statements (two conspiracy statements and two trivia statements had ranges of four as opposed to five on the 1 to 6 scale). All descriptive statistics are available on the OSF repository.

4 In Béna et al. (2023), they used a binary truth rating variable (1 = false, 2 = true). We used a different scale here because a continuous scale allowed for us to generate matched statement sets for the trivia and conspiracy statements. Because we selected the statements based on the 1 to 6 truth rating scale, we adopt that scale in the main study, as well. Moreover, since Béna et al. analyzed the illusory truth effect based on the means (rather than proportions) for the binary truth rating variables, we do not anticipate that the change in scale will substantively affect the results. Further, meta-analytic evidence suggests that the magnitude of the illusory truth effect does not differ between dichotomous and continuous scales (Dechêne et al., 2010).

5 We conducted an a priori power analysis and examined the sample size of 282 participants in *Superpower* (Lakens & Caldwell, 2021) for a 2 (repetition: repeated or new) × 2 (materials: conspiracy or trivia statements) within-subjects ANOVA. We entered the following into the power analysis: (1) a common standard deviation of 1.13 (determined based on the pretest) and (2) a correlation of .70 (similar to that observed in Pillai & Fazio, 2024, which also measured the effects of repetition within-subjects). We entered means for repeated (conspiracy: 2.77, trivia: 3.18) and new (conspiracy: 2.61, trivia: 2.61) statements that would generate a Cohen’s *d*_z_ of .17 for the conspiracy statements (based on the effect for conspiracy statements in Béna et al., 2023) and a Cohen’s *d*_z_ of .65 for the trivia statements (based on the effect for trivia statements in Béna et al., 2023); the means for the new statements were informed by the pretest ratings. With a total sample size of 282 participants, we would have 99% power to detect the effect from the 2 × 2 ANOVA (partial η^2^ = .10) for the repetition * materials interaction from Béna et al. (2023; and 80% power to detect an effect of partial η^2^ as small as .03). Thus, we were sufficiently powered to detect a partial η^2^ of .03 or greater. Full results from the power analysis are on the OSF repository.

6 Béna et al. (2023) examined the four-way interaction between repetition, materials, conspiracy mentality, and cognitive reflection. We did not have any hypotheses regarding the four-way interaction, it was not significant in the original study, and these analyses would require an extremely large sample size (even larger than required for the three-way interaction). Thus, we elected to not examine or power for the four-way interaction and instead focus on the three-way interaction. The three-way interaction will reveal whether the magnitude of the illusory truth effect across different statement types varies based on level of conspiracy mentality or cognitive reflection.

7 Although not preregistered, we also examined the two-way interaction between cognitive reflection and conspiracy belief and three-way interactions between (1) cognitive reflection, conspiracy belief, and materials and (2) cognitive reflection, conspiracy belief, and repetition in predicting truth ratings. The three-way interaction between cognitive reflection, conspiracy belief, and statement type was statistically significant (*b* = .00, *SE* = .00, *p* = .014), *t*(20,940) = 2.47). The negative relationship between cognitive reflection and accuracy ratings was attenuated at higher levels of conspiracy belief, especially for trivia statements. These results are available on the OSF platform.

## Code availability

All code is available on the OSF repository: https://osf.io/b8hjk/?view_only=85807ad9bdf649078316a4284a3424ec
