# Supplementary material for: Does repetition increase perceived truth equally for conspiracy and trivia statements? A registered replication report
Source: Psychon Bull Rev. 2026 Jan 7;33(1):39. doi: 10.3758/s13423-025-02836-w (PMC12779699; doi:10.3758/s13423-025-02836-w)
Supplement: Supplementary file 1 — Supplementary file1 (DOCX 24 KB) [file 13423_2025_2836_MOESM1_ESM.docx]

**Dr. Adam Osth**

*“I am happy to accept your submission PBR-RR-24-005.R1 titled “Does repetition increase perceived truth equally for conspiracy and trivia statements?: A registered replication report” as the successful first stage of a Registered Report or Replication (RRR) publication in Psychonomic Bulletin & Review. As you know, we are now committed to publication of your results assuming that you follow the steps outlined below:

1. The cover letter must certify that the data for the registered experiment were collected after receiving approval from PB&R.
2. The completed experiment(s) must have been executed and analyzed in the manner approved here with any unforeseen changes in those approved methods and analyses clearly noted.
3. The manuscript must describe and justify all post-hoc analyses.

While PB&R will commit to publishing the results, review of the final submission may lead to comments that need to be addressed in revision. Our commitment is to the results, not to the discussion section.”*

**Response:** Thank you for supporting this work!

1. We certify that the data for the preregistered experiment were collected after receiving approval from PB&R (data collection began after *PBR-RR-24-005.R1* was accepted).
2. We certify that the experiment was executed and analyzed in the manner approved. Any additional post-hoc/exploratory analyses are clearly noted.
3. Any additional post-hoc/exploratory analyses are justified.

**Reviewer 1**

*“I would like to thank the authors for their thorough revision and for addressing my initial comments. I am very excited about this registered report and believe that it will make a substantial contribution to our understanding of the generalizability of the illusory truth effect. I believe that all my initial comments from 2 onwards have been sufficiently addressed. I appreciate the thoroughness of the responses.”*

**Response:** Thank you for your helpful input!

*“On my first comment, I don’t think it’s necessary to run an additional pre-test or to match on other moments of the distributions in defining the sets. However, given that matching on the average plausibility of two sets with pre-tests conducted at different times is a noisy way to control for plausibility, I would like to see a robustness check on the main analyses including controls for the item-level plausibility. I’m happy with however you would like to include that in the analyses but am, myself, partial to a multi-level model.”*

**Response:** We agree that this analysis could be a helpful robustness check. In the Supplemental Materials document, we provide additional information surrounding the approach and the results from these secondary analyses. We also now note on p. 12 of the Results:

“In secondary analyses that were not pre-registered, we used mixed-effects regressions to examine the effects of repetition, statement, and their interaction on truth ratings for item-level truth ratings with participant and item random intercepts. These results are described in detail in the “Supplemental Materials” document on the OSF repository (Supplemental Materials 1; Supplemental Figure 1). To summarize, we found a very small, but significant, interaction between repetition and materials (*t*(20,940) = 2.01, *p* = .044, *b* = .08, SE = .04, 95% [.00, .15]) such that the magnitude of the illusory truth effect was slightly higher for trivia statements than for conspiracy statements.”

We additionally have added interpretation of these results in the Discussion on p. 18:

“Note that in exploratory mixed-effects regressions we did find a small interaction between repetition and materials. Nevertheless, even in the mixed-effects regressions, which accounted for item-level variability, the effect size was still small. Based on these results in total, when eliminating confounds due to differences in baseline plausibility, the effect of repetition on perceived truth is similar for conspiracy statements and false trivia statements.”

*“I would also like to see clarification of how you will interpret a null effect on the main interaction of interest and what effect sizes you can rule out with the amount of power that you have. Unless you are very highly powered to rule out even small differences in effect sizes (my interpretation of the partial eta^2 sizes you’re powered to detect is that they still may be meaningful but partial eta^2 is not a statistic that I deal with), I would like you to clarify your interpretation of a null result as something along the lines of “we can rule out differences in the effect of repetition between the sets of X% of the overall effect” or some other easily interpretable difference in effect sizes.”*

**Response:** We have addressed this helpful comment in three ways. First, we have included a follow-up Bayesian repeated measures ANOVA to compute a Bayes Factor for the repetition-by-materials interaction (pp. 13-14 of the Results). We now write: “We found weak evidence against including the interaction term (BFinclusion = .58). The data were 1.72 times more likely under the models without the interaction term than with the interaction term. Thus, these analyses provide additional evidence that there is not a meaningful repetition-by-materials interaction.” (p. 14 of the Results).

Second, to more directly address your comment we have added a clarifying statement on p. 12 of the Results section regarding our power analysis (Footnote #5). We now write: “With a total sample size of 282 participants, we would have 99% power to detect the effect from the 2 X 2 ANOVA (partial η^2^ = .10) for the repetition * materials interaction from Béna et al., 2023 (and 80% power to detect an effect of partial η^2^ as small as .03). Thus, we were sufficiently powered to detect a partial η^2^ of .03 or greater. Full results from the power analysis are on the OSF repository.”

Finally, in the Limitations subsection of the Discussion section, we describe that our study was underpowered to detect the identified interaction effect. We write on p. 19:

“First, we were not sufficiently powered to detect an interaction between repetition and materials as small as the observed effect of η^2^ = .01; post-hoc sensitivity analyses using *Superpower* in R revealed that we only had 41% power to detect the identified moderation effect. However, even if the interaction effect was statistically significant in a well-powered study, it is unlikely to be practically significant. The semantic, psychological, and plausibility differences between conspiracy statements and trivia statements do not strongly or substantively alter the effects of repetition on belief.”
